# Awareness of Cervical Cancer, Risk Perception, and Practice of Pap Smear Test among Young Adult Women of Dhulikhel Municipality, Nepal

**DOI:** 10.1155/2023/6859054

**Published:** 2023-08-26

**Authors:** Ishwori Byanju Shrestha, Sandesh Bhusal, Manish Rajbanshi, Prajita Mali, Rakhi Byanju Shrestha, Devendra Raj Singh

**Affiliations:** ^1^Department of Public Health, Asian College for Advance Studies, Purbanchal University, Lalitpur, Nepal; ^2^Research and Development Division, Dhulikhel Hospital, Kathmandu University, Kavre, Nepal; ^3^Nepal Health Frontiers, Tokha-5, Kathmandu, Nepal; ^4^Institute of Medicine, Tribhuvan University, Maharajgunj, Kathmandu, Nepal; ^5^Kathmandu University School of Medical Science, Kavrepalanchowk, Dhulikhel, Nepal; ^6^School of Human and Health Sciences, University of Huddersfield, Huddersfield, UK

## Abstract

**Background:**

Despite being one of the most preventable forms of cancer, cervical cancer remains an important public health problem, especially in developing countries. However, there is limited evidence regarding awareness and practice of screening for cervical cancer among women in resource-poor settings like Nepal. This study is aimed at assessing the awareness of cervical cancer, risk perception, and practice of Pap smear tests among adult women of Dhulikhel municipality of Kavreplanchowk district in Nepal. *Methodology*. A community-based cross-sectional study was conducted among 422 women (aged 18-45 years) residing across the Dhulikhel municipality of Nepal. Systematic random sampling method with face-to-face interviews was conducted to collect data. A descriptive analysis was performed to assess the sociodemographic characteristics of the participants. The chi-square test was used to determine the factors associated with risk perception and participants' demographic characteristics.

**Results:**

The mean age (±SD) of the participants was 30.7 ± 7.9 years. This study found that around 55% and 38% of women had heard about cervical cancer and Pap smear test, respectively. Of those who had heard of the Pap test, only 37.6% had ever practiced the test. Similarly, 33.2% and 12.1% knew about the correct age group and time interval to perform the Pap test, respectively. Among those who had heard about cervical cancer, nearly 57% had positive perceptions toward cervical cancer. In addition, risk perception of cervical cancer was found to be associated with participant age, family type, and marital status.

**Conclusion:**

The women had inadequate knowledge and practice of cervical cancer and Pap smear test. This study concluded the need for a context-specific and effective health awareness program to promote preventive measures for cervical cancer and enhance the practice of Pap smear test in the community.

## 1. Introduction

Globally, cervical cancer is the 4th most frequent malignancy in women, with an estimated 604,000 new cases and 342,000 deaths in 2020 [1]. It is the second most common cancer in women in the South-East Asia Region (SEAR) and becoming a major cause of cancer deaths among women in low and middle-income countries (LMICs) including Nepal [2].

Cervical cancer develops in a woman's cervix, and almost all cervical cancer cases (99%) are associated with high-risk human papillomavirus (HPV) infection. This virus frequently spreads through sexual contact. Although the majority of HPV infections are self-limiting and symptomless, persistent infections can lead to cervical cancer in females [3].

Despite being one of the most preventable and treatable forms of cancer, it continues to be a major cause of women killing 11 in 100000 women in developing countries after breast cancer [4]. In Nepal, cervical cancer is the most common cancer, with 1,493 annual deaths in 2020 [5]. The Government of Nepal (GoN) developed national guidelines for Cervical Cancer Screening and Prevention (CCSP) in 2010 with the goal of screening at least 50% of the target population (women aged 30–60 years). Also, the government provides free cervical screening in primary health centers through visual inspection with acetic acid (VIA) method and recommends screening every 5 years [6]. Despite the efforts, limited improvement has been observed reducing the cases of cervical cancer in Nepal. Women in rural areas of Nepal are particularly at higher risk for developing cervical cancer [7]. Delays in performing screening, women's illiteracy, financial constraints for health checkups, marriage at a young age, poor access to healthcare, and feeling shyness toward screening for cervical cancer in the community were often linked with the detection of cervical cancer among women residing in the semiurban or rural areas often [7–9].

The World Health Organization (WHO) has implemented a global strategy to accelerate the elimination of cervical cancer as a public health problem by achieving a 90-70-90 target by the year 2030 that has focused on HPV vaccination, screening, and treatment [10]. Despite WHO's targeted aim of reducing the incidence to less than four per 100,000 women, Nepal has approximately four times the incidence of cases compared to the global target indicating the public health concern of cervical cancer [11].

Awareness of cervical cancer, along with the sociocultural context of Nepal and its linkage of health-seeking behavior and practices, is critical to women's participation in the prevention and screening of cervical cancer among women. However, appropriate public health programs on cervical cancer have not been focused on and targeted to the vulnerable community. Still, a major proportion of women had poor practice with Pap smear tests, especially among women with low educational and economic status [11, 12]. These groups are more prone to developing cervical cancer due to the poor knowledge and screening practices in Nepal [4]. Also, there is limited evidence to inform policymakers and program designers for the prevention of cervical cancer in Nepal.

Therefore, this study is aimed at assessing knowledge of cervical cancer, risk perception, and practice of Pap smear tests among adult women of Dhulikhel municipality in Nepal. The findings provide insights into the perception of women on cervical cancer, awareness status of cervical cancer, and utilisation of cervical screening. The updated evidence from this study can inform the concerned stakeholders in designing interventions to reduce the incidence and mortality of cervical cancer in Nepal.

## 2. Methodology

### 2.1. Study Design and Setting

A community-based cross-sectional study was carried out from May to October of the year 2019. Dhulikhel municipality, one of the three municipalities of the Kavre district, in the central region of Nepal, was selected as a study site. Dhulikhel is one of the ancient cities of Kavre, located about 30 kilometers east of Kathmandu, the capital of Nepal, with a total population of 39, 047 as per census 2011.

### 2.2. Study Population and Sampling

The study population consisted of young adult females aged 18-45 years across the selected wards of Dhulikhel municipality. Participants having disabilities such as hearing, mental, or speech problems were excluded from the study.

Among the total of 12 wards of the municipality, two wards (wards 6 and 7) were selected. During the study period, there were a total of 905 households (375 in Ward 6 and 530 in Ward 7) in the selected wards as per the records of the municipality office. Systematic random sampling was conducted to select the household; only one eligible participant was selected from each household. If more than one eligible participant was found in the house, a simple random technique was used to select one participant.

The sample size was calculated using the following formula: *n* = *z*^2^pq/*e*^2^. Assuming the 50% prevalence, 5% margin of error, 95% confidence interval, and 10% nonresponse rate, the final sample size was determined as 422.

The sampling interval was calculated as 905/422 = 2.14. Therefore, the households were selected after every two houses.

### 2.3. Study Tools

A structured questionnaire was designed by referring to the available information from previous studies [13–15]. The questionnaire consisted of four sections including sociodemographic characteristics, perception regarding cervical cancer, behavioral risks, and practice of Pap smear tests. Sociodemographic characteristics include age, religion, ethnicity, marital status, educational level, family type, and occupation of the participants.

Questions on awareness of cervical cancer included cause, signs and symptoms, prevention, and treatment of cervical cancer. Perception toward cervical cancer was measured with 14 statements graded on a three-point Likert scale (agree, neutral, and disagree). Evaluation of perception was done by assigning “1” for a positive response and “0” for a negative response and neutral response. Median value was calculated from participants' responses, and values above the median score were regarded as positive perception.

Regarding the Pap smear test, six questions were related to the knowledge, which includes awareness of cervical cancer, source of information, the purpose of the test, the procedure of the test, appropriate age, and the right interval of the Pap smear test. The practice of Pap smear tests includes the availability of the test in the nearest institutions, the place of the test performed, and the reason for performing and not performing the test.

### 2.4. Data Collection

A structured questionnaire was used to collect data from participants. The principal investigator herself was involved in the data collection procedure and was assisted by research assistants. Before the data collection, research assistants were provided with an orientation by the principal investigator regarding the project and study tools. Face-to-face interviews were conducted in participants' households by trained research assistants. It took about 30 minutes to complete the interview.

### 2.5. Data Management and Analysis

Collected data were entered, compiled, coded, and cross-verified in the Epi-data version 3.1 software. Then, the data was exported to Statistical Package for the Social Sciences (SPSS) version 20 (IBM) for analysis. Data analysis consisted of descriptive statistics, including frequency distribution and percentages. In addition, a chi-square test was done to assess the association between risk perception and practice of Pap smear test with sociodemographic variables. All the tests were conducted at a significance level of 95% confidence interval.

### 2.6. Ethical Approval

The ethical approval for the study was taken from the Nepal Health Research Council (NHRC) (Ref no:3586). Permission was taken from the municipality office. Verbal and written consent was taken from each participant after explaining the objectives of the study before the data collection.

## 3. Results

### 3.1. Sociodemographic and Economic Characteristics of the Study Participants

A total of 422 participants were recruited for this study. The mean age (±SD) of the participants was 30.7 (±7.9), (range: 18-45) years in this study. The majority of the participants (73.2%) belonged to the age group of 25 and more years. Most of the participants (82.2%) were Hindu followed by Buddhist (10.2%). Almost three-fourths of the participants (73.6%) were Janajati followed by the Brahmin/Chhetri (16.6%) ethnic group. Similarly, around 61% were from joint/extended family, and 79.6% were married. Regarding the occupation of the participants, 44.8% were housewives followed by business (22.7%). Almost two-thirds (64.7%) of the participants had formal education (Table 1).

### 3.2. Source of Information on Cervical Cancer

**Figure 1:**
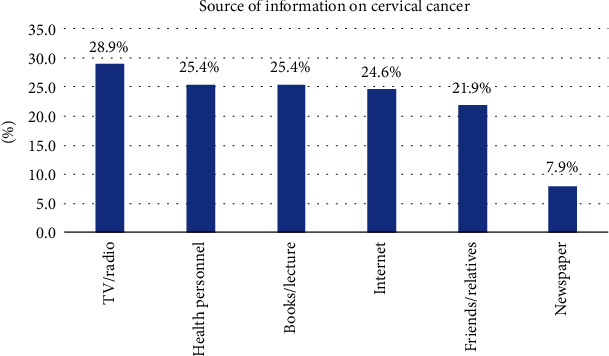
Sources of information on cervical cancer.

Among 422 participants, 229 (54.3%) have heard about cervical cancer (Table 2). The majority of the participants (28.9%) have heard about cervical cancer from TV/radio followed by health personnel (25.4%), books/lectures (25.4%), internet (24.6%), friends/relatives (21.9%), and newspaper (7.9%) (Figure 1).

### 3.3. Awareness of Study Participants on Cervical Cancer

Among 229 participants who have heard about cervical cancer, 137 (59.8%) have heard about the causes of cervical cancer. Having more than one sexual partner, having unsafe sex, and having poor genital hygiene were the major causes of cervical cancer responded by participants. Similarly, half of the participants (113 (49.3%)) knew about the signs and symptoms of cervical cancer. Among them 89.4% responded that unpleasant vaginal discharge was a major sign and symptom.

Among the 218 participants who felt cervical cancer could be prevented, 187 (85.7%) mentioned timely screening as a preventive method. Likewise, 209 (91.2%) participants believed it could be treated. Most of them (75.2%) believed that it could be treated by hysterectomy (Table 2).

### 3.4. Perception on Cervical Cancer among the Study Participants (*n* = 229)

**Figure 2:**
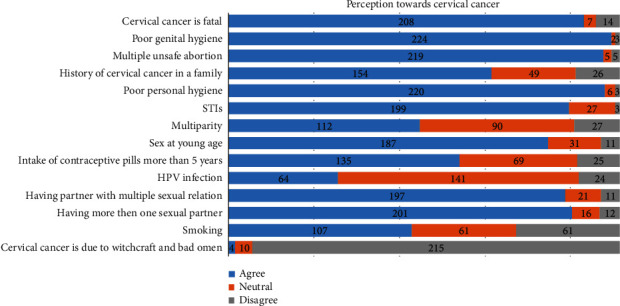
Perception toward cervical cancer among the participants.

Altogether, 14 statements were used to determine the perception of cervical cancer among the women. The perception score obtained by the participants ranges from 4 to 14 with the median score of 11 (IQR: 10-13). Around 57% of the participants were found to have positive perception toward cervical cancer (Figure 2).

### 3.5. Associated Factors toward the Perception among the Study Participants

Table 3 depicts that participant's age, family type, and marital status were significantly associated with perception of cervical cancer.

### 3.6. Knowledge of Pap Smear Test among the Study Participants

Only 37.2% of the participants had heard of the Pap smear test. Most of the participants had heard of Pap smear test from health personnel (45.2%) followed by books/lectures (22.3%). Among those who had heard about the Pap test, 88.5% correctly responded as a Pap smear test is done to detect cervical cancer. Similarly, 73.2% correctly answered the procedure for Pap smear test that cells from the cervix are scraped away and then test under microscope for abnormal growth. While only 33.2% of the participants were aware that a Pap smear test is done to the females who are 21 years and sexually active. Similarly, only 12.1% of the participants correctly answered that a Pap smear test is done every 3 years (Table 4).

### 3.7. Practice of Pap Smear Test among the Study Participants

A total of 157 and 59 participants had heard and performed Pap smear test, respectively. Among the participants, 144 (91.7%) had access to Pap smear test facilities in their nearest health institutions. Among those who had performed a Pap test, the majority (54.2%) performed the test in community hospital. The main reason for the Pap smear test among the participants was routine checkups (44.1%) followed by gynecological problems (27.1%). More than third-fourth (77.7%) of the participants who had not had a Pap smear test believed they were not at risk of cervical cancer followed by the unaware of Pap smear test in this study. (Table 5).

## 4. Discussion

This study assessed the knowledge, perception, and practice of Nepalese women regarding cervical cancer and Pap smear test.

In this study, around 55% of the participants have heard about cervical cancer which is comparable to the findings of the study conducted in Ethiopia [14] and North Korea [16]. Studies conducted in Qatar [17] and Niger [18] showed higher proportions (85% and 72%, respectively) of the participants who have heard of cervical cancer; however, studies in South Africa [19] and Ghana [20] showed that only 42.9% and 30.6% of the participants had heard of about cervical cancer, respectively. The differences in the proportions of participants who have heard about cervical cancer in the studies conducted in various countries could be due to factors like cultural and societal factors, education level, and healthcare infrastructures.

Mass media like TV and radio were the major source of information in line with the study's findings in North West Ethiopia [14]. The contrasting finding was reported in a study conducted in North Korea and Nigeria where healthcare providers were the primary source of information [16, 21]. This difference might be due to differences in media coverage between the countries. Another possible reason could be fear and embarrassment that discourage women to discussing cervical cancer with healthcare workers [17].

In this study, only 137 (59.8%) of the participants who had heard about it claimed they knew the causes of cervical cancer. Among them, having multiple sex partners, unsafe sex, and poor genital hygiene were mentioned as major causes for the cervical cancer that is comparable to studies conducted in Nepal, Saudi Arabia, and Ethiopia [15, 22, 23]. Most of the participants believed that unpleasant vaginal discharge is one of the major warning signs of cervical cancer similar to studies conducted in Nepal [24] and Saudi Arabia [23]. People of this community were aware due to the frequent running of awareness programs on cervical cancer by the local municipality and nongovernment organizations.

Among the participants who had heard of cervical cancer, more than 90% believed that cervical cancer is preventive and curable, which is similar to the study conducted in Qatar [17]. Screening on time and safe sexual practice were the major response as a preventive method for cervical cancer. However, a study conducted in North Korea and North West Ethiopia revealed that the majority of respondents did not know that cervical cancer can be prevented [14, 16]. This inconsistency in results between the studies might have emerged from the inadequate knowledge of participants between the countries.

We found that around 57% of the women had a positive perception toward cervical cancer which is comparable to the finding of studies conducted in South India [25], Saudi Arabia [26], and Ethiopia [27]. However, a study conducted by Tilahun et al. among female students found only 44% of them had positive attitudes toward cervical cancer preventive practices [22]. The results vary across the study, but the overall attitude toward cervical cancer risks and screening practices is inadequate among the women. In our study, participants in the higher age group had higher odds of having a positive perception of cervical cancer than the lower age group. Similar results were observed in other studies conducted in Ethiopia and the United Kingdom [22, 28]. Women of higher age may have had personal experiences as well as more exposure to information and education about cervical cancer over their lifetime, leading to increased knowledge and positive attitudes.

Similarly, women in nuclear families were more likely to have a positive perception of cervical cancer in comparison with joint or extended families. The possible explanation for this could be that the women in nuclear families may have more access to education, better access to healthcare services, and information about the importance of cervical cancer screening and prevention, leading to better practices [29]. Also, they may have more privacy and autonomy in making personal health decisions, which may increase the likelihood of them following preventive practices for cervical cancer. In our study, married women demonstrated more positive perception of cervical cancer than unmarried ones. This is consistent with the findings from other studies [28, 30, 31]. It is possible that married women may have a partner who provides emotional support, financial support, and encouragement to seek preventive health services.

Regarding screening for cervical cancer, only 37.2% of the total participants had heard about Pap smear tests. Similar results were observed in the previous study conducted in Kathmandu, Nepal [13]. The uptake of Pap smear test (12.1%) was less in our study participants. This outcome indicates that besides the lack of information regarding the Pap smear test, the majority of those who were familiar with it were unaware of how frequently or how many times they should undergo the test during their lifetime. The lower uptake of Pap smear tests might also be due to the beliefs in local myths about cervical cancer, stigmatization of women with cervical cancer, misconceptions about the causes of cervical cancer, and the lack of spousal support for cervical cancer screening [32]. It can also be attributed due to a lack of screening advocacy and a national screening program.

This study provides insights into women's knowledge and preventive practices toward cervical cancer and attributable demographic characteristics, which could be useful in designing educational programs on cervical cancer screening and prevention. This study used a locally pretested and validated tool. As this study is conducted in community settings using random sampling, results are more generalizable in other similar community settings in resource-limited settings like Nepal.

## 5. Conclusion

Overall, the study results indicated that women's awareness or level of knowledge, perception, and screening practices toward cervical cancer is unsatisfactory. Imparting knowledge of cervical cancer through various awareness campaigns and also by the health personnel during their visits to the health center for any other reasons would improve uptake of the screening tests. Thus, the study highlighted the need for improvement in health literacy and implementing health promotion programs and campaigns regarding the cause, risk factors, and early screening of cervical cancer.

## Figures and Tables

**Table 1:** Characteristics of the study participants.

Variables	Frequency	Percentage
Age in years		
Mean ± SD	(30.7 ± 7.9)	
25 years and above	309	73.2
Below 25 years	113	26.8
Religion		
Hindu	347	82.2
Buddhist	43	10.2
Christian	26	6.2
Muslim	6	1.4
Ethnicity		
Janajati	311	73.6
Brahmin/Chettri	70	16.6
Dalit	34	8.1
Others	7	1.7
Family type		
Joint/extended	257	60.9
Nuclear	165	39.1
Marital status		
Married	336	79.6
Unmarried	86	20.4
Occupation		
Housewife	189	44.8
Business	96	22.7
Student	58	13.7
Government/private service	56	13.3
Others	23	5.5
Educational level		
Illiterate/informal	149	35.3
Formal	273	64.7
Family history of cervical cancer		
No	415	98.3
Yes	7	1.7
No of children		*N* = 304
1-3	292	96.1
More than 3	12	3.9

**Table 2:** Awareness of cervical cancer among the study participants.

Questions	Frequency	Percentage
Heard about cervical cancer (*n* = 422)	229	54.3
Know about the cause of cervical cancer (*n* = 229)	137	59.8
Causes of cervical cancer^∗^ (*n* = 137)		
Having more than one sexual partner	92	67.2
Having unsafe sex	92	67.2
Poor genital hygiene	82	59.9
Partner having more than one sexual relation	55	40.1
Having sex at young age	45	32.8
STIs	41	29.9
Having multiple unsafe abortions	39	28.4
Smoking	37	27
Intake of birth-controlling pills more than 5 years	35	25.5
HPV	28	20.4
Gynecological problems	12	8.7
Others	7	5.1
Know about the sign and symptoms of cervical cancer (*n* = 229)	113	49.3
Signs and symptoms of cervical cancer^∗^ (*n* = 113)		
Unpleasant vaginal discharge	101	89.4
Persistent lower back pain	51	45.1
Intermenstrual bleeding	44	38.9
Vaginal bleeding after menopause	39	34.5
Vaginal bleeding during and after sex	36	31.9
Stomachache	27	23.9
Others	6	5.3
Can cervical cancer be prevented (*n* = 229)	218	95.2
Preventive method of cervical cancer^∗^ (*n* = 218)		
Timely screening for cervical cancer	187	85.7
Safe sexual practice	94	42.9
Avoid smoking	46	21
Avoid early marriage and pregnancy	34	15.5
Maintaining personal and genital hygiene practice	33	15.1
HPV vaccination	30	13.7
Timely treatment of STIs	30	13.7
Use of condom while having sex	28	12.8
Do not know	7	7
Others	5	2.3
Can cervical cancer be treated (*n* = 229)	209	91.2
Treatment methods of cervical cancer^∗^ (*n* = 209)		
Hysterectomy	158	75.2
Chemotherapy	66	31.4
Medication	55	26.2
Pelvic exenteration	28	13.3
Radiotherapy	22	10.5
Do not know	13	6.2

^∗^Multiple responses. Abbreviations: STIs: sexually transmitted infections; HPV: human papillomavirus.

**Table 3:** Factors associated with the perception of cervical cancer among the study participants.

Variables	Perception category		*P* value	Odds ratio (95% CI)
	Positive *n* (%)	Negative *n* (%)		
Level of perception	130 (56.8)	99 (43.2)		
Age				
25 years and above	103 (60.9)	66 (39.1)	0.03^∗^	1.9 (1.1-3.5)
Below 25 years	27 (45.0)	33 (55.0)		
Family type				
Nuclear	79 (62.7)	47 (37.3)	0.04^∗^	1.7 (1.0-2.9)
Joint/extended	51 (49.5)	52 (50.5)		
Education level				
Formal education	120 (58.5)	85 (41.5)	0.19	1.9 (0.8-4.5)
Illiterate/informal education	10 (41.6)	14 (58.4)		
Marital status				
Married	107 (73.8)	38 (26.2)	<0.001^∗∗^	7.4 (4.0-13.6)
Unmarried	23 (26.4)	61 (72.6)		
Occupation				
Housewife	52 (54.7)	43 (45.3)	0.6	0.8 (0.5-1.5)
Nonhousewife	78 (58.2)	56 (41.8)		

^∗^ *p* value less than 0.05. ^∗∗^*p* value less than 0.001 level.

**Table 4:** Knowledge toward the Pap smear test among the study participants.

Variables	Frequency	Percentage
Heard about Pap smear test (*n* = 422)		
Yes	157	37.2
No	265	62.8
Source of information^∗^ (*n* = 157)		
Health personnel	71	45.2
Books/lectures	35	22.3
Friends/relatives	33	21.0
TV/radio	32	20.4
Internet	20	12.7
Newspaper	12	8.3
Pap smear is done to (*n* = 157)		
To detect cervical cancer	139	88.5
To treat cervical cancer	2	1.3
To treat STIs	1	0.6
Do not know	15	9.6
Procedure during Pap smear test (*n* = 157)		
Cells from cervix are scraped away and then test under microscope for abnormal growth	115	73.2
Blood is tested for cervical cancer	1	0.6
Cervix of women is taken out for testing	3	1.9
Do not know	38	24.3
Appropriate age for performing Pap smear test (*n* = 157)		
Below 21 years	12	7.6
21 years and are sexually active	52	33.2
Immediately after having first sex	23	1.9
Do not know	90	57.3
Right interval for Pap smear test (*n* = 157)		
Every 3 years	19	12.1
Every 5 years	6	3.8
Every year if you have more than one risk factors	39	24.8
No need to further test if the first Pap smear test is negative	2	1.3
Do not know	88	56.1
Others	3	1.9

^∗^Multiple responses.

**Table 5:** Practice of Pap smear test among the study participants.

Practice item	Frequency	Percentage
Availability of Pap smear test in nearest health institution (*n* = 157)		
Yes	144	91.7
No	6	3.8
Do not know	7	4.5
Ever had a Pap smear test (*n* = 157)		
Yes	59	37.6
No	98	62.4
Place of test performed (*n* = 59)		
Community hospital	42	54.2
Health camps	13	23.7
Private clinic	9	15.3
Governmental hospital	4	6.8
Result of the test (*n* = 59)		
Positive	2	3.4
Negative	57	96.6
Reason for performing test^∗^ (*n* = 59)		
Being at risk	2	3.4
Suggestion of a doctor	14	23.7
Check-up routine	26	44.1
Gynecological problems	16	27.1
Others	2	3.4
Reason for not performing Pap smear test^∗^ (*n* = 98)		
Not being at risk	80	77.7
Unaware of Pap smear test	17	16.6
Expensive	2	1.9
Shy toward Pap smear test	2	1.9
Others	2	1.9

^∗^Multiple responses.

## Data Availability

Data are available on reasonable request to the corresponding author.
